# Children's biobehavioral reactivity to challenge predicts DNA methylation in adolescence and emerging adulthood

**DOI:** 10.1111/desc.12739

**Published:** 2018-09-21

**Authors:** Sarah J. Goodman, Danielle S. Roubinov, Nicole R. Bush, Mina Park, Pau Farré, Eldon Emberly, Clyde Hertzman, Marilyn J. Essex, Michael S. Kobor, W. Thomas Boyce

**Affiliations:** ^1^ Centre for Molecular Medicine and Therapeutics BC Children's Hospital Research Vancouver BC Canada; ^2^ Medical Genetics University of British Columbia Vancouver BC Canada; ^3^ Psychiatry University of California San Francisco California; ^4^ Pediatrics University of California San Francisco California; ^5^ School of Population and Public Health University of British Columbia Vancouver BC Canada; ^6^ Physics Simon Fraser University Burnaby BC Canada; ^7^ Human Early Learning Partnership University of British Columbia Vancouver BC Canada; ^8^ Psychiatry University of Wisconsin School of Medicine and Public Health Madison Wisconsin; ^9^ Child and Brain Development Program Canadian Institute for Advanced Research Toronto ON Canada

## Abstract

A growing body of research has documented associations between adverse childhood environments and DNA methylation, highlighting epigenetic processes as potential mechanisms through which early *external* contexts influence health across the life course. The present study tested a complementary hypothesis: indicators of children's early *internal*, biological, and behavioral responses to stressful challenges may also be linked to stable patterns of DNA methylation later in life. Children's autonomic nervous system reactivity, temperament, and mental health symptoms were prospectively assessed from infancy through early childhood, and principal components analysis (PCA) was applied to derive composites of biological and behavioral reactivity. Buccal epithelial cells were collected from participants at 15 and 18 years of age. Findings revealed an association between early life biobehavioral inhibition/disinhibition and DNA methylation across many genes. Notably, reactive, inhibited children were found to have decreased DNA methylation of the *DLX5* and *IGF2* genes at both time points, as compared to non‐reactive, disinhibited children. Results of the present study are provisional but suggest that the gene's profile of DNA methylation may constitute a biomarker of normative or potentially pathological differences in reactivity. Overall, findings provide a foundation for future research to explore relations among epigenetic processes and differences in *both* individual‐level biobehavioral risk and qualities of the early, external childhood environment.

## Graphical abstract

This study examined whether children's early internal, biological, and behavioral responses to stress were predictive of DNA methylation later in life. Findings revealed an association between early life biobehavioral reactivity and DNA methylation in multiple genes measured at both 15 and 18 years of age. As such, DNA methylation may constitute a biomarker of normative or potentially pathological differences in reactivity.

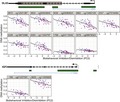

**RESEARCH HIGHLIGHTS**

- Measures of temperament, presyndromal mental health symptoms, and autonomic stress reactivity were input into principal component analysis (PCA) to create a composite measure distinguishing inhibited children from disinhibited children.
- 93 DNA methylation sites measured at age 15 were significantly different between inhibited and disinhibited children, including nine sites located within the *DLX5* homeobox gene and two sites in *IGF2*, a growth factor.
- *DLX5* and *IGF2* DNA methylation at age 18 was also significantly correlated with childhood inhibition/disinhibition.

## 1 INTRODUCTION

From the earliest moments of life, children's health and development are shaped by the qualities of their environmental contexts. Processes termed ‘biological embedding’ elucidate the possible mechanisms of such relations and describe how exposures to environmental adversity get ‘under the skin’ to influence critical biological pathways affecting health across the lifespan (Boyce & Kobor, 2014; Hertzman & Boyce, 2010). Epigenetic processes represent one model of biological embedding and have been increasingly recognized as a potential link between stressful childhood environments and later health outcomes (Boyce & Kobor, 2014; LaSalle, Powell, & Yasui, 2013). DNA methylation (DNAm) patterns associated with environment or experience are also influenced by other factors such as individual health behaviors, differences in temperament, and disease states (Ng et al., 2012).

DNAm is the most studied epigenetic modification in human populations and consists of a methyl group addition to the 5′ cytosine of CpG dinucleotides (CpGs). Once believed to be a gene silencing epigenetic mark, DNAm is context‐ and location‐specific and has been linked to increased, decreased, and unchanged gene activity (Edgar, Tan, Portales‐Casamar, & Pavlidis, 2014; Gutierrez‐Arcelus et al., 2013; Jones, 2012). The complex mechanisms by which DNAm can alter gene activity include inhibiting or enhancing transcription factor binding to DNA, recruiting enzymes to alter histone modifications, and altering splice sites, among others (Jones, 2012; Yin et al., 2017). DNAm is most dynamic during fetal development when epigenetic patterns play an integral part in the complex processes of embryogenesis (Guo et al., 2014; Khavari, Sen, & Rinn, 2010) and rates of change generally stabilize in adulthood (Alisch et al., 2012). However, adolescence is also understood to be a time of increased methylome alterations (Alisch et al., 2012; Lister et al., 2013), though studies of DNAm changes during this developmental period are more scarce compared to those conducted in early childhood and later adulthood (Jones, Goodman, & Kobor, 2015).

A growing body of research has revealed associations between exposures to early life environmental and psychosocial adversity and DNAm in accessible tissues such as buccal epithelial cells (BECs), saliva and peripheral blood (for an excellent review of the epigenetics patterns of traumatic stress, see Vinkers et al. (2015)). For example, children reared in institutional environments show increased DNAm among many genes in peripheral blood mononuclear cells (PBMCs) and whole blood, as compared with children reared by biological parents (Esposito et al., 2016; Naumova et al., 2012). DNAm measured in tissues including PBMCs, saliva, BECs also appears to be associated with early life experiences of low socioeconomic status (Lam et al., 2012), childhood maltreatment or deprivation (Klengel et al., 2012; Kumsta et al., 2016; Non et al., 2016; Weder et al., 2014) and maternal mental health problems during the perinatal period (Hompes et al., 2013; Monk, Spicer, & Champagne, 2012). In a prior study of this cohort conducted by our research team, exposure to maternal stress in infancy and childhood was associated with differential DNAm among offspring in mid‐adolescence (Essex et al., 2011). Paternal stress in childhood was also associated with DNAm changes in mid‐adolescence among female offspring only.

Beyond the influence of adverse early environmental experiences, epigenetic patterns may also be shaped by intra‐individual biology. Genetic variation, for example, is a strong predictor of DNAm patterns (Bell et al., 2011; Chen et al., 2016; Fraser, Lam, Neumann, & Kobor, 2012). Allelic variation may alter individual susceptibility to adverse social and environmental conditions leading differences in DNAm (Meaney, 2010). For example, an allelic variant of the *FKBP5* stress‐response gene altered whether adults who experienced childhood abuse or trauma also exhibited loss of DNAm at this gene (Klengel et al., 2012). In addition to allelic variation, factors indexing an individual's internal psychological and physiological state may also be associated with patterns of DNAm (Conradt et al., 2015; Ouellet‐Morin et al., 2013).

Empirical studies examining the association between individual‐level phenotypic factors and epigenetic differences are scarce. In two papers, measures of physiological reactivity to stress during infancy and childhood were associated with DNAm of BECs and placental cells (Conradt et al., 2015; Ouellet‐Morin et al., 2013), and physical aggression in early life has been shown to predict differential patterns of DNAm in T cells in adulthood (Guillemin et al., 2014; Provençal et al., 2014). Recent research in a group of young rhesus macaques also showed anxious temperament to be associated with differentially methylated loci in the central nucleus of the amygdala (Alisch et al., 2014).

A limited number of studies have thus examined associations among discrete biological and behavioral stress response parameters, psychological health, and epigenetic modifications. The inherent coupling involved in ‘mind‐body relations’, however, suggests that a more comprehensive understanding might be gleaned from synthesizing the interrelations among individuals’ internal, individual‐level biological and behavioral qualities into an integrated factor that could be examined for associations with DNAm. To this end, the current study derived measures of children's biobehavioral response predispositions from the shared variation among temperamental traits, presyndromal behavioral symptoms, and autonomic reactivity to stressful laboratory challenges. Temperament has been defined as ‘constitutionally based individual differences in reactivity and self‐regulation in the domains of affect, activity, and attention’ (Rothbart & Derryberry, 1981). Such differences have established biological underpinnings and are known to influence children's physiological and behavioral responsivity to environmental conditions (Goldsmith et al., 1987; Kagan, 2012; Rothbart, 1989). Both temperament and stress reactivity are related to the development of later forms of psychopathology and may act as antecedent, subclinical precursors, or endophenotypes (Caspi, Moffitt, Newman, & Silva, 1996; Muris & Ollendick, 2005; Pine & Fox, 2015; Rutter, 1984). When taken together, an integrative measure of temperamental traits, presyndromal mental health symptoms, and biological reactivity might plausibly provide a more powerful indicator of a child's internal biobehavioral response predispositions than if those domains are explored independently. To our knowledge, no extant research has examined relations between biobehavioral responses and DNAm over time.

In light of previous research, the present study examined prospective associations between early, *internal* differences in biobehavioral responses and later epigenetic modifications across two time points within a sample of 55 individuals from the Wisconsin Study of Families and Work (WSFW). This developmentally oriented, longitudinal research project established a birth cohort from which data on child temperament, autonomic reactivity, mental health, and DNA were collected at multiple time points from prenatal life to age 18. We anticipated significant relations between childhood biobehavioral response propensities (i.e., internal factors) and adolescent patterns of DNAm, paralleling our prior work (and that of other investigators) documenting linkages between stressful life experiences (i.e., external, environmental factors) and DNAm. We examined the relations between early life biobehavioral measures and DNAm at two time points, 15 and 18 years. Utilizing these rich longitudinal data, we ran an additional analysis of the temporal stability of DNAm, examining whether such stability is required for the longitudinal persistence of biobehavioral associations.

## 2 METHODS

### 2.1 Study sample

Participants in the current study were drawn from a WSFW subsample (*n* = 120) of children, parents, and teachers (Hyde, Klein, Essex, & Clark, 1995). Children were selected for that subsample to provide a balanced representation of high and low reported mental health symptoms (Boyce et al., 2001). The present analyses are based on a subset of 55 children who had complete data on temperament, mental health symptomatology, and ANS reactivity in the infancy, preschool, and kindergarten periods, and who provided DNA samples at ages 15 and 18 years (Table 1). Mann–Whitney *U* tests indicated no significant differences on any biological or behavioral measure between the 55 children in the present analysis and the larger WSFW subsample from which they were drawn (*p* > 0.05 at each variable). Of the 55 individuals, 19 were male and 36 were female. Mean family income measured at 12 months postpartum and preschool (4.5 years old) was $51,480 (median = 47,000) and $63,220 (median = 56,000), respectively. Six children were of non‐Caucasian minority status. All children entered primary school in the same school year (in 1996). Ethics approval for the WSFW was obtained from the University of Wisconsin‐Madison Institutional Review Board and informed consent was obtained from all participants.

**Table 1:** Mental health, temperament and ANS traits collected over 7 years and included in the analysis. Variable names signify reporter first, time point second and measure last

Parameter	Variable	Reporter	Age/time point	Instrument	Measure	Min.^a^	Median^a^	Max^a^
ANS response	OG1‐HR	Observational	Grade 1	ANS stress reactivity	Heart rate reactivity (slope)	−1.30	−0.23	2.85
	OG1‐PEP				Pre‐ejection period reactivity (slope)	−2.34	0.41	1.13
	OG1‐RSA				Respiratory sinus arrhythmia reactivity (slope)	−2.31	0.29	1.20
	OG1‐MAP				Mean arterial pressure reactivity (slope)	−1.42	−0.22	1.97
Temperament	MI‐AN	Mother	12 months	IBQ	Approach negativity (activity level, distress to limitations)	−2.14	−0.02	1.99
	MI‐WN				Withdrawal negativity (distress to novelty, startle)	−2.09	0.00	3.06
	MP‐WN		Avg. 3.5 & 4.5 years	CBQ	Withdrawal negativity (fear, sadness, shyness)	−2.36	0.03	1.74
	OP‐WN	Observational	4.5 years	LabTAB		−2.31	−0.10	1.85
	OG1‐WN		Grade 1			−2.58	−0.08	2.01
	MP‐ANG	Mother	Avg. 3.5 & 4.5 years	CBQ	Approach negativity (anger)	−2.48	0.10	1.87
	OP‐ANG	Observational	4.5 years	LabTAB		−1.67	0.14	2.34
	OG1‐ANG		Grade 1			−1.99	−0.09	1.81
Mental health symptom	MK‐INT	Mother	Kindergarten	HBQ	Internalizing (depression, separation anxiety, overanxious)	−1.34	−0.19	2.82
	TK‐INT	Teacher				−0.85	−0.43	3.61
	MK‐EXT	Mother			Externalizing (oppositional, conduct, overt aggression)	−1.67	−0.10	2.59
	TK‐EXT	Teacher				−0.57	−0.45	3.85

Note

M: mother‐report; T: teacher‐report; O: observed; I: infancy; P: preschool; K: kindergarten; G1: grade 1; AN: approach negativity; WN: withdrawal negativity; ANG: anger; INT: internalizing symptoms; EXT: externalizing symptoms; HR: heart rate; PEP: pre‐ejection period; RSA: respiratory sinus arrhythmia; MAP: mean arterial pressure.

a Reporting the standardized descriptives that were used for PCA.

### 2.2 Measures

A summary of all measures collected at each time point, organized by construct, can be found in Table 1.

#### 2.2.1 Autonomic nervous system reactivity

During in‐home assessments completed during first grade, children participated in a 15‐min standardized, developmentally appropriate stress reactivity protocol (Alkon et al., 2002). Briefly, the protocol consisted of challenges across social (interview with the child), cognitive (digit recall task), sensory (a taste identification task), and emotional (a fear‐ and sadness‐eliciting movie clip) domains (Alkon et al., 2002). Measurements of ANS activity, including heart rate (HR), pre‐ejection period (PEP), respiratory sinus arrhythmia (RSA), and mean arterial pressure (MAP) were assessed via electrocardiography and impedance cardiography throughout the protocol (Alkon et al., 2002; Boyce et al., 2001). Autonomic reactivity was indexed as increases in HR and MAP and decreases in PEP (reflecting sympathetic activation) and RSA (reflecting parasympathetic withdrawal), relative to resting levels (Boyce et al., 2001). All specific reactivity measures were computed as the slope of the ANS measure reactivity regressed on time. Positive slopes on HR and MAP and negative slopes on RSA and PEP all indicated upregulation in general ANS arousal (Boyce et al., 2001).

#### 2.2.2 Temperament

Temperament was assessed via both maternal report and observational coding methods, capturing the specific domains of temperamental negativity that have been theoretically and empirically related to children's physiological reactivity (Gray, 1991). First, mothers reported on the Infant Behavior Questionnaire (IBQ; Gartstein & Rothbart, 2003) at age 1 year and a modified version of the Child Behavior Questionnaire (CBQ; Rothbart, Ahadi, Hershey, & Fisher, 2001) at ages 3.5 and 4.5 years. Both instruments have been widely validated for use in their respective target populations (Gartstein & Rothbart, 2003; Parade & Leerkes, 2008; Putnam & Rothbart, 2006; Rothbart et al., 2001).

Observational measures of children's temperament were also collected using the Laboratory Temperament Assessment Battery (LabTAB) administered in a standardized fashion during home assessments at 4.5 years and in grade one. The LabTAB is comprised of 12 emotion‐eliciting behavioral episodes that simulate everyday situations (e.g., a social interaction with an unfamiliar adult, waiting for a signal before eating a snack, and using fine motor skills at a toy workbench). These situations were used to evoke affective reactions within three domains: negative affectivity, positive affectivity, and behavioral control‐regulation. All LabTAB assessments were videotaped and rated by two independent reviewers who coded facial, vocal, motor, behavioral, and postural responses (Gagne, Van Hulle, Aksan, Essex, & Goldsmith, 2011). To remain consistent with the temperament domains assessed by maternal report on the IBQ and CBQ, only coded behavioral observations of approach negativity and withdrawal negativity, elicited during negative affectivity episodes, were included in the present analyses. Maternal report and laboratory‐based observations of temperament and behavior that were not expected to relate to children's physiological reactivity were excluded. For additional details of LabTAB episodes, administration, and coding (see Luby et al., 2002). Both LabTab‐ and questionnaire‐derived temperamental measures have been shown reliable and internally consistent in this sample (Gagne et al., 2011).

#### 2.2.3 Mental health symptoms

Children's presyndromal, internalizing, and externalizing symptoms were assessed in kindergarten using maternal and teacher reports on subscales from the MacArthur Health and Behavior Questionnaire (HBQ) (Boyce et al., 2002; Essex et al., 2002). The HBQ internalizing and externalizing subscales are well‐validated measures of emotion regulation and reactivity difficulties relevant to the present analyses of children's biobehavioral reactivity (e.g., sadness, withdrawal, irritability, anger)(Lemery‐Chalfant et al., 2007; Luby et al., 2002). The validity and reliability of these measures have been previously established in this sample (Luby et al., 2002).

#### 2.2.4 DNA methylation

##### Extraction and bisulfite conversion of DNA from buccal swabs

Buccal epithelial cells were collected from participants at ages 15 and 18 years using MasterAmp Buccal Swabs (Epicentre Biotechnologies) and were stored at ‐80º C. Genomic DNA was extracted from buccal swabs using Buccal DNA Isolation Kits (Isohelix Ltd), then purified and concentrated with DNA Clean & Concentrator kits (Zymo Research). DNA quality was assessed by a NanoDrop ND‐1000 (Thermo Scientific). 750 ng of genomic DNA underwent bisulfite conversion using the EZ DNA Methylation Kit (Zymo Research).

##### Microarray experiments

Bisulfite converted DNA was treated according to established protocols (Illumina) in preparation for loading onto microarrays. DNA from buccal swabs collected at age 15 years was assayed using the Infinium HumanMethylation27 BeadChip (27K array); previous findings on these data can be found in Essex et al. (2011). DNA from samples collected at age 18 was assayed using the next generation of this technology, the Infinium HumanMethylation450 BeadChip (450K array).

For samples collected at age 15, 160 ng of bisulfite‐converted DNA was whole‐genome amplified, fragmented, and hybridized onto the 27K array. The 27K array analyzes DNAm at 27,578 CpGs, primarily at DNA sequences that map onto gene promoter regions. Raw DNAm data from the scanned microarrays are available in the gene expression omnibus (GEO) database under the accession number GSE25892 at: https://www.ncbi.nlm.nih.gov/geo/query/acc.cgi?acc=GSE25892 (Essex et al., 2011).

For samples collected at age 18, 160 ng of bisulfite‐converted DNA was whole‐genome amplified, fragmented, and hybridized onto the 450K array. The 450K array covers over 485,000 CpGs, representing 99% of all RefSeq genes, and includes ~90% of the CpGs that are on the 27K array. Following scanning of the microchips, data were input into Illumina's Genome Studio software.

##### DNAm data processing

For the 27K array, DNAm data were background‐adjusted and quantile‐normalized, as previously described (Essex et al., 2011). Probes underwent rigorous quality control processes, and were assessed for detection *p*‐value, number of underlying probes, and signal levels in each subject and replaced with ‘NA’ in the subject of interest if they failed at any metric (total of 4567 CpG measurements). One sample was removed based on poor quality data (>10% probes with a detection *p* > 0.05), leaving 109 samples, and one probe was removed due to poor quality data (detection *p* > 0.05 in more than 10% of individuals). The remaining samples had between 0 and 194 ‘NA’ values (mean = 27.19, median = 13), which were replaced with imputed values using the ‘impute.knn’ function in the R package ‘impute’ (Troyanskaya et al., 2001). Probes on the X and Y chromosomes were removed as these CpGs differ by sex. Finally, probes were removed based on lack of inter‐individual variability in accordance with existing data reduction methods. First, probes in which β values across all individuals were <0.05 or >0.95, were excluded (9,346) (Bourgon, Gentleman, & Huber, 2010). Second, analyses were restricted to individuals with complete biobehavioral measures only (*n* = 55) and any probes with a range <0.05 β, as calculated between individuals within the 10th and 90th percentile were removed (8,309). This left 9,922 probes for analysis (Lemire et al., 2015). This variability cut‐off is stringent but reasonable, as most probes measured on the 27K array are located within promoter CpG islands, which are the most invariable CpGs in the genome (Edgar et al., 2014). The 9,922 CpGs were annotated to 6,583 unique genes; 2,212 genes contained two or more variable probes. Finally, the ‘detectOutlier’ function in the lumi package was applied and no outliers were identified (*n* = 55) (Du, Kibbe, & Lin, 2008).

DNA methylation data from the 450K array were background‐adjusted and color‐corrected in Genome Studio (Illumina). Three outlier samples were detected using the ‘detectOutlier’ and removed, leaving 52 individuals. A total of 4,314 probes deemed low quality were removed. Additional probes that were removed included those that mapped onto sex chromosomes (1, 216), assayed single nucleotide polymorphisms (SNPs; 64), or were found to cross‐hybridize to sex or autosomal chromosomes (37, 909) or SNPs (19, 999), based on a previous annotation (Price et al., 2013). Data were then normalized across samples using quantile‐normalization, followed by normalization of probe‐type differences using Subset‐quantile Within Array Normalization (SWAN) (Maksimovic, Gordon, & Oshlack, 2012). An empirical Bayes method (ComBat) was applied to correct for effects associated with the separation of samples into batches during the microarray experiments, specifically into 96‐well plates, into microarray chips holding 12 samples per chip, and into six rows present on each chip (W. E. Johnson, Li, & Rabinovic, 2006).

Of note, the DNAm data collected at ages 15 and 18 were run at different times and on different technologies (the 27k array and the 450k array, respectively), and these data were treated differently during preprocessing. Specifically, the 450k array contains two probe types (type I and type II), which have different dynamic ranges and therefore different β value distributions; this was corrected for using SWAN. The 450k array was also corrected for batch effects using ComBat due to technical variability. The 27k array contains only type I probes and batch effects as measured by plate, chip and row were not significantly correlated with our variables of interest (all *p* > 0.05), nor were they correlated with the first principal component of the DNAm array data, which accounted for over 90% of total variability. Therefore, neither SWAN nor ComBat were used on the 27k array data. For consistency, CpG sites assayed on both the 27k array and the 450k array were mapped to genes using the Price annotation, while the Illumina HumanMethylation27 Manifest File was used for CpG assayed only on the 27 array (Price et al., 2013).

At each CpG assayed on the microarray platforms, a β value ranging from 0 (completely unmethylated) to 1 (100% methylated) was calculated for each sample using the signal intensity from the scan. β values of all samples from the 27K array and 450K array were log transformed into M‐values prior to analysis to adjust for the heteroscedastic nature of β values (Du et al., 2010). All results are reported in β values to facilitate biological interpretation.

### 2.3 Statistical analysis

#### 2.3.1 Principal component analysis

Principal component analysis (PCA) is a multivariate technique designed to reduce the dimensionality of a large set of non‐independent variables, while retaining important sources of variation in the dataset (Ringnér, 2008). In the present analysis, PCA was used to derive biological and behavioral response propensities that reflect the shared, underlying regulatory processes common across the measures of temperament, ANS reactivity, and internalizing/externalizing symptoms (Figure S1). PCA provided an effective analytic alternative to conducting separate analyses of the relations between the biological/behavioral measures and DNAm, which would have been statistically and conceptually problematic due to the increased rate of Type I error and the interrelatedness of our reactivity measures. PCA was run on the 16 scaled variables derived from measures of temperament, ANS, and internalizing/externalizing symptoms (Table 1), producing 16 principal components (PCs) that represent mathematically unique but conceptually overlapping aspects of the child's biological and behavioral response propensities. Each PC (hereafter referred to as a *biobehavioral reactivity factor*) represents an independent axis of variation among the data, driven by different combinations of weights from the original measures. The first three biobehavioral reactivity factors were chosen for DNAm analysis based on examination of the scree plot, which indicated a clear ‘break’ between the third and fourth component (Bro & Smilde, 2014).

#### 2.3.2 Covariates

A number of covariates were evaluated for potential inclusion in the analyses. The sample was homogenous in age (all participants entered preschool within the same school year) and ethnicity (87% Caucasian), precluding the need to control for these variables. Family income, measured at 9 months postpartum and kindergarten, were also excluded as covariates, because they were not correlated with any of the three biobehavioral PCs (all *p* > 0.05). The effect of sex was examined *post hoc*.

The cell composition of buccal swabs can vary between individuals, altering DNAm patterns, and was therefore also considered for inclusion as a covariate. The percentages of underlying BECs and leukocytes were calculated from the DNAm profiles measured at age 15 using a cell deconvolution algorithm trained on BECs and saliva samples (Smith et al., 2015). Using this tool, the proportions of BECs in our age 15 cohort was estimated to range from 81% to 96% (mean 87.7%). However, this proportion was not correlated with our measures of interest (*p* > 0.05) and was also excluded from further analysis.

#### 2.3.3 Associations between biobehavioral reactivity and DNAm

##### Age 15

Associations between the biobehavioral reactivity factors and DNAm at age 15 were examined from the 27K array. The sample of 9,922 variable CpGs was tested against the three biobehavioral reactivity factors using Spearman rank order correlations. *p*‐values were corrected using the Benjamini‐Hochberg method to estimate false discovery rates (FDRs), which limits the expected proportion of false positives and therefore reduce the number of Type I errors (Benjamini & Hochberg, 1995). The magnitude of change in DNAm across individuals, termed Δβ, was calculated using the slope of the regression line and reported for each differentially methylated CpG as a measure of effect size (Lam et al., 2012). CpGs that were significantly associated at a FDR corrected *p*‐value of 0.05 or smaller and had an absolute Δβ >0.05 were reported as high confidence differentially methylated CpGs (Essex et al., 2011). CpGs with a FDR corrected *p*‐value between 0.05 and 0.2 and an absolute Δβ >0.05 were reported as medium confidence differentially methylated (Essex et al., 2011).

##### Persistence of associations between biobehavioral reactivity and DNAm at age 18

To test whether associations identified at age 15 persisted at age 18, we examined associations between the biobehavioral reactivity factors and DNAm measured at age 18 using the 450K array. Because age was perfectly confounded with both microarray platform and processing methods, we chose not to compare directly β values at ages 15 and 18 and rather repeated the correlations using DNAm assessed at age 18. This tested whether variation across individuals was also associated with biobehavioral reactivity at the later time point. As described in more detail below, we focused on genes with multiple high and medium confidence CpGs found at age 15. Again, *p*‐values were corrected using the Benjamini‐Hochberg methods to control for FDR. As an additional test of the strength of the associations, correlation coefficients between the PCA‐derived biobehavioral reactivity factors and differentially methylated CpGs were permuted 100 times, generating a null distribution, and compared to the true correlation coefficients.

#### 2.3.4 Longitudinal stability of DNAm

We also tested stability in DNAm from age 15 to age 18 using mixed effects models that evaluated the prediction of DNAm at age 18 from DNAm at age 15. This analysis was performed on a subset of the CpGs significantly associated with biobehavioral reactivity at age 15. As such, biobehavioral reactivity was included as a covariate.

#### 2.3.5 GO analysis

Gene ontology (GO) analysis was performed using the software ErmineJ (Gillis, Mistry, & Pavlidis, 2010; Lee, Braynen, Keshav, & Pavlidis, 2005). The 9,922 CpG sites used in the age 15 analysis were annotated to genes as previously described, in order to generate a complete gene list or ‘background’ from which to test for enrichment (Price et al., 2016). Enrichment analysis was then performed using precision‐recall and the following parameters: use the best scoring replicate, include only ‘Biological Process’ related GO terms, minimum gene set size of 5, maximum gene set size of 100, and test the effect of multifunctional genes.

#### 2.3.6 Pyrosequencing experiments

Pyrosequencing experiments were performed in order to validate significant associations between children's biobehavioral reactivity factors and DNAm in the *DLX5* gene found at both age 15 and age 18. A pyrosequencing assay was designed to examine the *DLX5* 3′ CpG island; this assay spanned approximately 200 base pairs and included five CpGs, two of which are covered by the 450K array (cg12041387, cg08835113); cg12041387 is also covered by the 27k array. Of the 55 child samples used in this analysis, only 42 had enough remaining genomic DNA from samples collected at age 18 to conduct experiments. All reactions were run on a PyroMark Q96 MD Pyrosequencer, following the manufacturer's protocol. All CpG loci passed Pyro Q‐CpG software quality control. Primer sequences used for DNA amplification and pyrosequencing are available upon request.

## 3 RESULTS

### 3.1 Principal components analyses of biobehavioral reactivity

**Figure 1:**
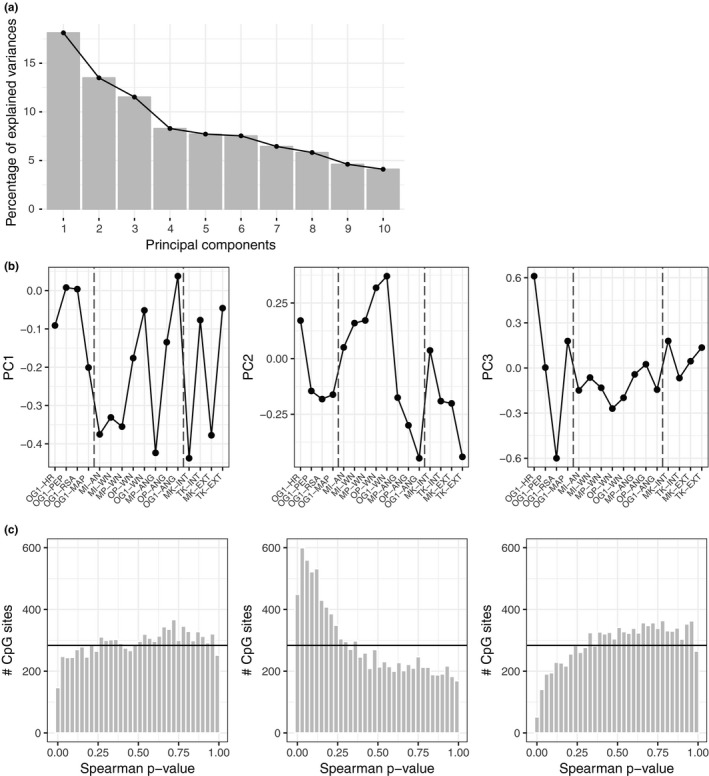
Results of principal component analysis revealed biobehavioral reactivity as biologically driven composite measure. (a) Plotting the percent variability of the principal components (PCs) showed a flatter distribution than what is typically expected when running PCA on psychological variables.(b) Loadings of original variables onto PC1, PC2 and PC3 (from left to right). (lc) *p*‐value distributions of genome‐wide correlations between DNA methylation and PC 1–3 (left to right) (*n* = 55)

Following PCA, the first three principal components were examined to derive and understand the primary factors that described children's biobehavioral reactivity. The first PC explained approximately 18% of the total variation, while the second and third PCs explained 13.5% and 11.5%, respectively **(**Figure 1a**)**.

Maternal report of children's temperament and behavior loaded strongly onto the first PC, while teacher report and observational measures of children's biobehavioral reactivity did not (Figure 1b). Thus, the first PC distinguished mothers’ broad perspectives on children's functioning from measures of children's reactivity in more context‐ and stressor‐specific settings. The third PC differentiated HR reactivity (an indicator of the activity of both the parasympathetic and sympathetic branches of the ANS) from RSA (a measure of parasympathetic activity only; Figure 1b). Our review of the PCA results indicated that both the first and third principal components reflected variance attributable to method‐ and/or reporter‐based differences, and thus we hypothesized that these components would not associate with DNA methylation (DNAm).

### 3.2 Associations between biobehavioral reactivity factors and DNAm at age 15

As expected, when correlated with DNAm at all 9,922 CpGs measured at age 15, PC1 and PC3 displayed uniform *p*‐value distributions, suggestive of a null distribution, and neither PC was significantly correlated with any individual CpG after FDR correction (Figure 1c).

The second PC represented individual differences in children's biobehavioral inhibition and disinhibition, ascertained across teacher‐, maternal‐, and laboratory‐based observational measures **(**Figure 1b**)**. Observed measures of withdrawal negativity and internalizing symptoms loaded positively onto PC2, whereas anger and externalizing symptoms loaded negatively. Measures of autonomic reactivity also showed moderate loadings on PC2: HR loaded positively, while PEP, MAP and RSA loaded negatively. Thus, PC2 integrated both behavioral and biological response characteristics—across reporters, contexts, and stressors—distinguishing inhibited children (higher scores) from disinhibited children (lower scores) and is hereafter referred to as Biobehavioral Inhibition/Disinhibition (BID). Correlations between BID and DNAm showed a left‐skewed *p*‐value distribution that deviated from the distribution that would be expected by chance, suggesting an association with age 15 DNAm **(**Figure 1c**)**. Examining this association more closely, we found that BID was significantly associated with 12 CpGs at an FDR cut‐off of 0.05 and an absolute Δβ >0.05 or 5% (‘high confidence’ CpGs; Table S1) and an additional 81 CpGs were associated with at an FDR between 0.05 and 0.2 and an absolute Δβ >0.05 (‘medium confidence’ CpGs). Therefore, this multi‐method, multi‐reporter composite trait of early life biobehavioral reactivity showed an observable and statistically significant DNAm signature. To check the robustness of our findings, a linear regression was also run on all 9922 CpGs, using BID as the explanatory variable and including sex and minority status as covariates. The significant CpGs were largely stable across statistical tests (Figure S2). Given the lack of covariate effects and our interest in the basic bivariate associations, we focus on the results of the Spearman correlations

Next, genomic locations of the 93 high confidence and medium confidence CpGs differentially methylated by BID were examined. These CpGs mapped to multiple genes, including the imprinted genes, GNAS complex locus (*GNAS*) and insulin‐like growth factor 2 (*IGF2*), and genes related to neurotransmitter secretion, including vesicle‐associated membrane protein 5 (*VAMPS*) and otoferlin (*OTOF*) (Table S1). However, after running gene ontology analysis on genes ranked by *p‐*value of associated CpGs, we did not find that genes containing differentially methylated CpGs could be classified by shared biological processes.

**Figure 2:**
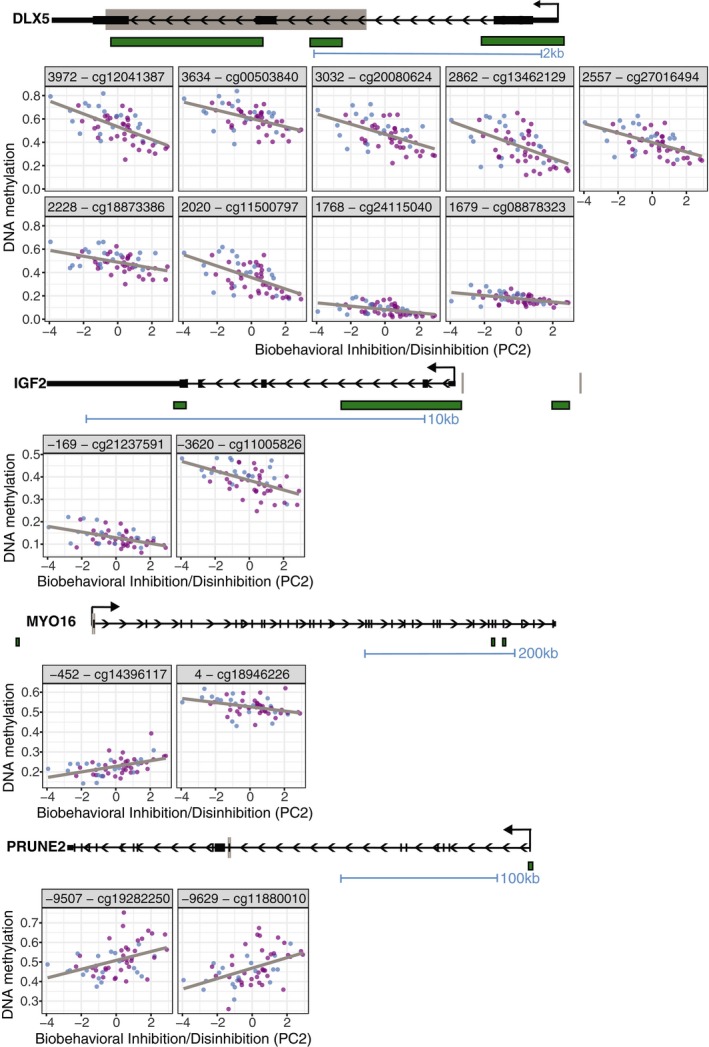
Schematic of *DLX5*,*IGF2*,*MYO16* and *PRUNE2* genes, which each contained more than two CpGs significantly associated with Biobehavioral Inhibition/Disinhibition. Green bars in gene schematic represent CpG Islands and gray lines or boxes represent genomic locations of CpGs plotted. Scatter plots of individual CpG DNA methylation are colored by sex (males = blue, females = pink) (*n* = 55)

All genes contained only a single differentially methylated, high or medium confidence CpG, with the exception of four: *DLX5* (distal‐less homeobox 5), *IGF2*,* MYO16* (myosin XVI), and *PRUNE2* (prune homolog 2) (Table S1). Nine CpGs mapped to the *DLX5* gene, located within the coding region of the gene between 1.5 and 4 kb downstream of the transcription start site. Five were high confidence CpGs (FDR < 0.05) and four were medium confidence (FDR 0.05–0.2) (Figure 2). The nine CpGs were contiguous except for one interrupting CpG that fell just outside of the medium confidence threshold (cg02101486, FDR *p* < 0.209), and all were highly correlated (Figure 2b). These CpGs thus constitute a differentially methylated region (DMR), a region in which multiple, adjacent CpGs exhibit associations with BID. All nine differentially methylated CpGs within *DLX5* correlated negatively with BID scores, with Spearman's rank order correlation coefficients (rhos) ranging between −0.60 and −0.44. The nine high and medium confidence CpG sites possessed large DNAm differences observed across individuals with the highest and lowest BID scores; the Δβ ranged from 9% to 38% (Michels, Binder, & Dedeurwaerder, 2013).

*IGF2*,* PRUNE2,* and *MYO16* each contained two probes significantly associated with BID. *IGF2* contained one high confidence CpG (cg11005826, FDR *p* < 0.04, rho = −0.52, Δβ = 15%) and one medium confidence CpG (cg21237591, FDR *p* < 0.13, rho = −0.45, Δβ = 9%), which were negatively associated with BID and were located 3,620 bp and 169 bp upstream of the transcript start site, respectively. *PRUNE2* contained two medium confidence CpG sites which were positively correlated with BID scores (cg11880010, FDR *p* < 0.09, rho = 0.47, Δβ = 18%; cg19282250, FDR *p* < 0.15, rho = 0.42, Δβ = 15%), located 9,620 and 9,507 bp upstream of the transcript start site, respectively. Finally, *MYO16* contained two medium confidence CpGs. One CpG, located 452 bp upstream of the transcription start site, was positively correlated with BID (cg14396117, FDR *p* < 0.08, rho = 0.48, Δβ = 10%) and one CpG, located 4 bp downstream of the transcription start site was negatively correlated (cg18946226, FDR *p* < 0.13, rho = −0.44, Δβ = 7%).

To ensure that effect sizes of the high and medium confidence sites were not being inflated by individuals with extreme DNAm values, correlations with BID were recalculated after a 90% winsorization (5% was modified from each tail) of all medium and high confidence CpGs. Results remained largely unchanged and the mean difference between *p*‐values generated before and after winsorization was 6.66 × 10^−6^ (median = −6.84 × 10^−7^, 1st quartile = −1.67 × 10^−5^, 3rd quartile = 1.63 × 10^−5^). Prior to winsorization nominal *p*‐values ranged from 1.06 × 10^−6^ to 1.97 × 10^−3^ and after winsorization nominal *p*‐values ranged from 9.52 × 10^−7^ to 2.1 × 10^−3^ (Table S2). Similarly, Δβ differences were minor; effect sizes changed by −4.05 × 10^−3^ on average after winsorization (median = −1.71 × 10^−3^).

To add an additional level of rigor to the analyses and to test that these correlations did not occur by chance, we permuted the BID scores 100 times and correlated the permuted scores with the 15 high and medium confidence CpGs mapping to *DLX5*,* IGF2, PRUNE2,* and *MYO16* (Figure S3 **)**. With the exception of cg14396117, all of the true correlation coefficients were significantly greater than correlations expected by chance (*p* < 0.01). The correlation coefficient for cg14396117, located in *MYO16*, fell outside the 99th percentile of the null distribution (*p* < 0.02). Therefore, our findings were unlikely to be spurious, but rather reflected significant associations between DNAm of *DLX5* and childhood reactivity, as measured by BID.

### 3.3 Examination of sex differences in correlations between biobehavioral reactivity factors and DNAm at age 15

**Figure 3:**
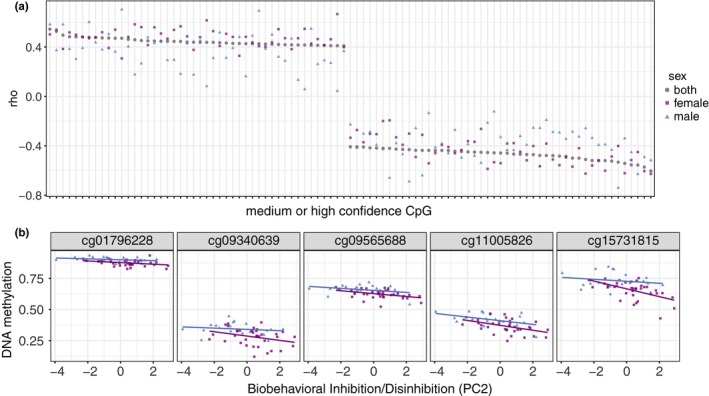
Results of correlations between DNA methylation at age 15 and Biobehavoural Inhibition/Disinhibition when cohort is separated by sex. (a) Spearman's correlation coefficients of 93 high and medium confidence CpGs, calculated in full cohort (gray circle, *n* = 55), females only (pink square, *n* = 36) and males only (blue triangle, *n* = 19). (b) Five CpGs which were associated with Biobehavioral Inhibition/Disinhibition and differentially methylated by sex. In all CpGs, correlations remained significant in females only but lost significance in males (*n* = 55)

Given previously observed differences between sexes in DNAm, temperament, and mental health symptoms, correlations between the 93 BID‐associated CpGs were reexamined separately within males (*n* = 19) and females (*n* = 36) (Singmann et al., 2015; Verhulst, van der Ende, Ferdinand, & Kasius, 1997). In general, the correlation coefficients (rhos) generated in females were similar to the coefficients generated when including both sexes in correlations (mean absolute difference in rho = 0.07) (Figure 3 top panel); however, the correlation coefficients generated in the males differed more strongly (mean absolute difference in rho = 0.13). To test whether this sex difference was driven by sample size, females were subsampled 100 times down to the sample size of males (*n* = 19) and correlations were rerun. The correlations were predominantly stable (mean absolute difference in rho = 0.02) (Figure S4), suggesting that the difference in sexes was not entirely driven by sample size.

**Figure 4:**
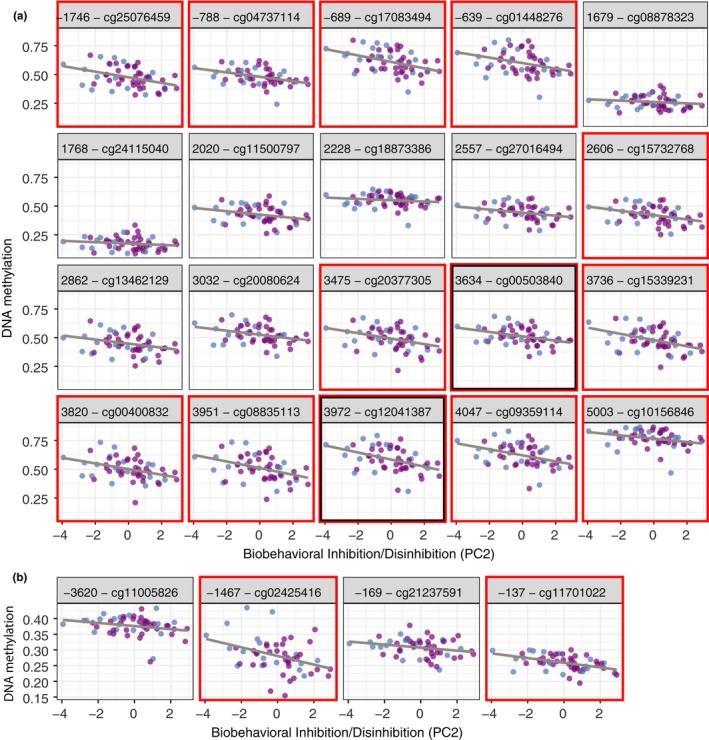
DLX5 and IGF2 DNA methylation remained significantly associated with Biobehavioral Inhibition/Disinhibition at age 18. (a) DNA methylation at age 18 in probes located upstream and within the DLX5 gene. (b) DNA methylation at age 18 in four probes located upstream of IGF2 gene. Panel titles represent distance from transcription start site, followed by CpG ID. Correlations found to be significant at age 18 are labeled with a red box; Correlations found to be significant at both age are labeled with a red/black box; all remaining CpGs were found to be significant at age 15 only. Males are plotted in blue; females are plotted in pink (*n* = 52)

Additionally, we directly compared the DNAm values of males to females and assessed whether high and medium confidence CpGs differed by sex. A Mann–Whitney *U* test was run on all 9,922 CpGs sites to test for CpGs differentially methylated by sex, revealing 192 such sites (FDR *p* < 0.05), including five CpGs significantly associated with BID (cg01796228, cg09340639, cg09565688, cg11005826, cg15731815). These mapped to the genes *LIFR*,* FCRL1*,* CLEC12B*,* IGF2*, and *RNF207*. In all five CpGs differentially methylated by sex, associations with BID remained significant in females (all *p* < 0.05) but were no longer significant in males (all *p* > 0.05) (Figure 3 bottom panel).

We then examined potential sex differences in biobehavioral reactivity‐DNAm relations within the *DLX5* gene only. All correlations with CpGs (9 CpGs) remained significant in females, with correlation coefficients ranging from −0.38 to −0.63 (*p* *< *0.05). In males, two CpGs, cg12041387 and cg11500797, remained significant, with correlation coefficients of −0.52 and −0.49, respectively (*p* < 0.05). In sum, only five of the 93 CpGs reported (cg01796228, cg09340639, cg09565688, cg11005826, cg15731815) were differentially methylated by sex, suggesting that DNAm patterns linked to BID are not likely driven by sex‐specific differences.

### 3.4 Persistence of associations between BID and DNAm at age 18

Given the strong associations between BID scores and multigenic DNAm at age 15 years, we hypothesized that those same associations would hold 3 years later, when participants were 18 years old. Using the 450K data at age 18, we examined all variable CpGs annotated to our four genes of interest to take advantage of the added coverage of the 450K array, as compared to the 27k array. We examined correlations between BID scores and 39 CpGs in *DLX5*, 35 in *IGF2*, nine in *PRUNE2,* and 37 in *MYO16*; this included all CpGs in those genes found to be significant at age 15.

A total of 15 probes at age 18 were found to be significant after FDR correction for the 120 tests (FDR *p* < 0.05 and Δβ > 0.05 or 5%) (Figure 4, Table S3). As before, these differentially methylated CpGs were permuted 100 times to create null distributions, and all observed correlation coefficients either fell outside of the 97% percentile of the null distribution (Figure S4 **)**.

The significant probes included 13 *DLX5* CpGs, including two of the nine probes found significant at age 15 (cg12041387 [rho = −0.45], cg00503840 [rho = −0.39]). An additional four CpGs that were significant at age 15 had an uncorrected *p* < 0.05 at age 18 but did not pass FDR correction (Spearman's rho = −0.34 to −0.31, Δβ = 10%–12%). There were an additional 11 significant CpGs at age 18 that were assayed only on the 450K array and thus not tested at age 15. These were located within the same region reported at age 15, as well as upstream of the transcription start site (600–1,700 bp). Again, effect sizes were notable and ranged from 10% to 21%.

The remaining two age 18 probes significantly associated with BID were *IGF2* CpGs (cg02425416 and cg11701022) with correlation coefficients of −0.40 and −0.47. These were not measured at age 15. Two *IGF2* CpGs measured at age 15 that were significantly associated with BID (cg11005826 and cg21237591) were no longer significant at age 18.

### 3.5 Longitudinal stability in DNAm

Taking further advantage of the longitudinal DNAm data, we directly examined its stability between the age 15 and 18. First, we compared the inter‐individual ranges of 17039 CpG sites run on both platforms, after removing low quality CpGs and those at which β values across all individuals were <0.05 or >0.95. A few sites differed substantially, an expected finding given that samples were taken 3 years apart. However, the average difference in inter‐individual variation neared zero, suggesting no systematic difference across our study sample due to either age or microarray platform (median = −0.011, mean = −0.022) (data not shown).

**Figure 5:**
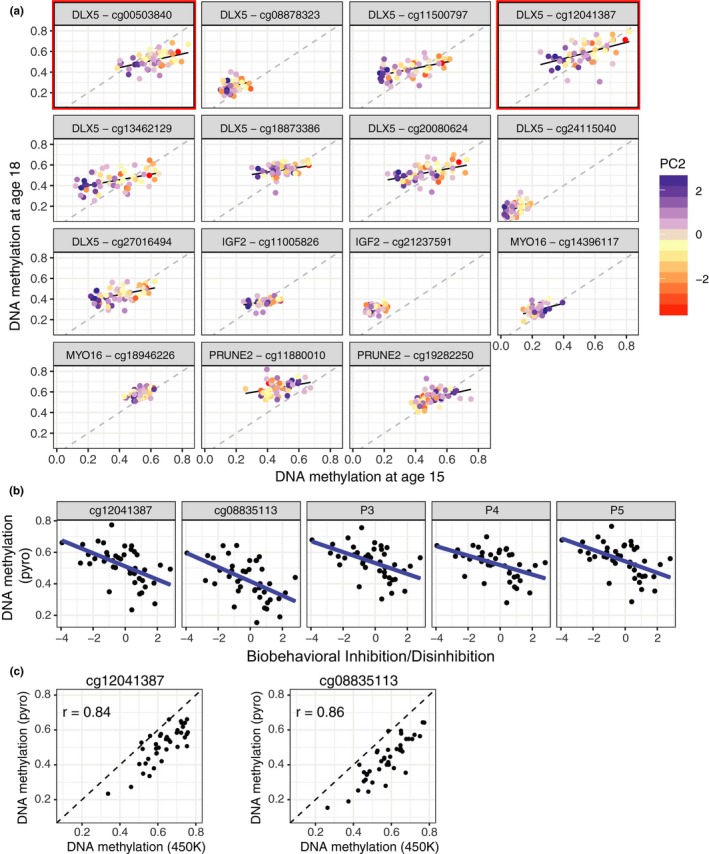
CpG stability across 3 years and pyrosequencing verification of *DLX5* gene. (a) Changes in DNA methylation in 15 CpGs measured across both ages. Red/black boxes indicate CpGs significantly associated with Biobehavioral Inhibition/Disinhibition at both ages; all remaining CpGs were associated at age 15 only (b) Five CpGs assayed by pyrosequencing DNA collected at age 18 were associated with Inhibition/Disinhibition scores. Two CpGs are identified by their 450K IDs, the remainder were not assayed by the 450K array. (c) Correlations of DNA methylation values at cg12041387 and cg08835113 generated by pyrosequencing and the 450K array (*n* = 42)

We then examined the 15 CpGs found in *DLX5*,* IGF2*,* PRUNE2,* or *MYO16*, that were significantly associated with BID at age 15, to ask whether these sites would reflect the global trend in stability seen above. Using a linear mixed effects model, DNAm at age 15 was tested as the explanatory variable, predicting DNAm at age 18, and BID was included as a covariate. These regression models were significant for 13 of the 15 CpGs (FDR corrected *p* < 0.05), with DNAm at age 15 explaining 8%–24% of the variation in DNAm at age 18 (Table S4). Although DNAm patterns at ages 15 and 18 were significantly associated, median DNAm in the 15 CpGs changed from −18% to +11%, with DNAm decreasing in 13 of the 15 CpGs 3 years later (Figure 5a). These findings suggested a lack of stability of DNAm from age 15 to 18.

### 3.6 Pyrosequencing verification of associations between BID and DLX5 DNAm

Finally, pyrosequencing experiments were conducted to verify the DNAm patterns in *DLX5* detected on the microarray platforms. All five probes in the pyrosequencing assay were negatively correlated with BID **(**Figure 5b), with correlation coefficients ranging from −0.47 to −0.52. However, DNAm levels assessed by pyrosequencing were consistently 5%–15% lower than in the 450K array in the two CpGs measured in both platforms (Figure 5c). Serial dilutions of artificially methylated and unmethylated samples run on pyrosequencing confirmed that the assay was not biased; this indicated that the array probes at these loci may exhibit a preference for binding methylated DNA over unmethylated DNA.

## 4 DISCUSSION

Current research on the epigenetic alterations that accompany early adversity exposures has led to a growing set of studies supporting *external,* environmental influences on DNA methylation (DNAm). Much of this research has examined the relation between early childhood stressors on DNAm (Anacker, O'Donnell, & Meaney, 2014; Boyce & Kobor, 2014). However, variations in physiological and psychological functioning have also been shown to correlate with DNAm (Alisch et al., 2014; Conradt et al., 2015; Guillemin et al., 2014) and may reflect the linkage of a child's *internal* reactivity to the environment through epigenetic signatures. Few empirical studies have tested this latter supposition, however, and those that have are mainly cross‐sectional in nature. We sought to address these limitations by testing the hypothesis that children's biological and behavioral response propensities would also be related to DNAm measured later in life. We also examined the persistence of relations between Biobehavioral Inhibition/Disinhibition (BID) and DNAm at age 15 and age 18 and the stability of DNAm at these CpGs.

Multiple‐reporter and multi‐method measures of early childhood temperament, behavior, and ANS reactivity were input into a PCA examining early life biobehavioral reactivity factors. Three components explained a significant percentage of the overall variance in temperament, mental health symptoms, and ANS reactivity and were further examined for their associations with DNAm at age 15. Two components, PC1 and PC3, were not associated with DNAm patterns at age 15, likely because they reflected method‐ and reporter‐based variance rather than trait‐like differences in children's biobehavioral reactivity. The second principal component (PC2), however—BID—reflected the intersection of observed temperamental withdrawal, anger, autonomic reactivity, and internalizing/externalizing symptoms and was found to have both a broad DNAm signature across many genes and a particularly strong association with multiple sites within *DLX5* and *IGF2* genes.

Elevated levels of children's biobehavioral *disinhibition* (approach negativity, anger, externalizing symptoms) were associated with significantly higher DNAm in *DLX5* and *IGF2* at age 15 and at age 18 years. Conversely, those with greater childhood *inhibition* (fear, withdrawal negativity, internalizing symptoms, and heart rate response) showed lower *DLX5* and *IGF2* methylation (Figure S6). While median DNAm within these CpGs changed over the course of 3 years, the association between children's early biobehavioral reactivity and *DLX5* and *IGF2* methylation was maintained from adolescence into young adulthood, a period marked by significant changes across various domains of psychological development (Arnett, 2000). This illustrates the paradoxically fixed yet dynamic nature of DNAm. Despite changes across the lifespan, there may be enduring patterns of DNAm in certain genes over time, particularly those associated with early biological and behavioral reactivity.

Given the tissue of origin in the current study (BECs), we can only suggest that these DNAm patterns represent a biomarker of behavioral reactivity. However, Gene‐Tissue Expression (GTEx) RNA sequencing data (Lonsdale et al., 2013) indicate that *DLX5* is expressed in skin tissue and some brain regions, and it is possible that the magnitude of inter‐individual DNAm differences in *DLX5* reflects actual differences gene transcription levels, affecting downstream biological processes (Michels et al., 2013). *IGF2* is not expressed at significant levels in either skin tissue or brain regions, though this does not negate a potential for DNAms differences to be a biomarker of biobehavioral reactivity. Importantly, results of present research are preliminary, and validation of these findings in a larger sample, with more repeated measures of DNAm and reactivity, are needed to advance understanding of a possible causal relation.

*DLX5* is homeobox gene involved in neuron, craniofacial, and bone development. Its protein regulates glutamic acid decarboxylases involved in the synthesis of gamma‐aminobutyric acid (GABA), the chief inhibitory neurotransmitter in GABAergic neurons (Stühmer, Anderson, Ekker, & Rubenstein, 2002). If the differences in DNAm observed in our cohort correspond to regulatory epigenetic patterns in brain tissue, such differences could produce altered levels of GABA, corresponding to inhibited or disinhibited behavioral proclivities. Although GO analysis of our CpGs ranked by *p*‐value did not find any significant enrichment of GABA‐related genes, these results cannot fully rule out the involvement of GABA circuitry. Future research should explore the underlying pathways between reactivity‐DNAm associations.

*DLX5* is also highly expressed in osteoblasts during embryogenesis and plays an important role in craniofacial development. Commensurate with our finding of temperament and behavioral response‐associated differences in *DLX5* methylation, aspects of temperament have been previously linked to the bizygomatic width of facial structure, i.e., the ratio of the facial diameter across the cheekbones to the vertical height of the head. Specifically, 4‐month‐old infants who showed propensities to biobehavioral reactivity had smaller bizygomatic widths (i.e., narrower faces) at 14 and 21 months than infants who were less reactive (Arcus & Kagan, 1995). Although the various functions of the *DLX5* gene suggest that the protein may act as a regulator of the early development of inhibition and reactivity, further research is needed to confirm the relation between inhibition and *DLX5* DNAm.

*IGF2* is expressed in many fetal tissues and encodes a growth factor that primarily acts to promote overall growth during gestation via cell differentiation and proliferation. Unlike the *DLX5* gene, the function of DNAm patterns in this gene has been thoroughly studied. *IGF2* is imprinted and expressed only from the paternal allele (Giannoukakis, Deal, Paquette, Goodyer, & Polychronakos, 1993). Aberrations in imprinting of this gene and other nearby genes are associated with Beckwith‐Wiedemann and Silver‐Russell syndromes—two congenital, growth‐affecting conditions—as well as a number of cancers (Choufani, Shuman, & Weksberg, 2013; Eggermann, 2009; Joyce et al., 1997; Tycko, 2000; Weksberg, Shen, Fei, Song, & Squire, 1993). While there are no published clinical features of Beckwith‐Wiedemann relating to temperament, parents of affected children often describe them as more tenacious than their siblings (R. Weksberg, personal communication, December 2017). Previous studies of *IGF2* methylation have also found associations with ADHD symptoms and prenatal maternal anxiety (Rijlaarsdam et al., 2017; Vangeel et al., 2015). Given that our BID measure was comprised of measures of internalizing and externalizing symptoms, these previous associations between *IGF2* methylation and mental health are commensurate with the identified relation between DNAm at this gene and BID.

The focus of this study was on early life internal, individual differences in biobehavioral reactivity in order to extend research that has largely focused on relations between DNAm and external, environmental adversities. However, the developmental influences of such internal and external factors do not operate in isolation of each other, as demonstrated by existing literature on environmental correlates of *IGF2* methylation. In a previous study of this cohort, associations were observed between early parental stress and DNAm in adolescence (see Essex et al., 2011). BID was not associated with DNAm levels in CpGs found related to parental stress; however, early parental stress was significantly associated with CpGs in *IGF2* and *DLX5*, although not those identified here. In sum, results of the present study provide a strong foundation on which future research can further explore relations among external environmental influences, internal biobehavioral factors, and DNAm patterns.

### 4.1 Limitations

Results of the present study must be weighed in light of several limitations. First, the current study's assessment of DNAm at ages 15 and 18 years suggests at least short‐term persistence of associations with biobehavioral reactivity, but we are unable to infer when differences in DNAm may have arisen in development. It is possible that the observed DNAm pattern was established during early embryogenesis in the ectodermal germ layer, in response to allelic differences and fetal exposures. If this were true, then both BECs and neurons, with their common ectodermal origins, might be expected to exhibit comparable patterns of DNAm. While it is possible that DNAm in tissues other than BECs played a causal role in the development of differing levels of biobehavioral reactivity, DNAm and reactivity may be related in at least two other ways. DNAm patterns could result from temperamental response predispositions leaving a chemical mark on the epigenome, or a third factor, such as genetics, could have affected both reactivity and DNAm. Regarding the latter, we did not test our CpGs of interest for the influence of genetic variability although such CpGs, termed methylation quantitative trait loci, are common in the genome (Bell et al., 2011; Teh et al., 2014). The relatively small and homogenous nature of our sample precluded extensive testing of the influence of race and sex on relations between BID and DNAm, though our results were retained across models that controlled for the effects of covariates. Future longitudinal studies with larger, more heterogeneous samples are needed to build on the present findings and address more complex questions of antecedence, causality, and modifying factors.

Due to ethical and other conspicuous prohibitions on acquiring samples of brain, we studied DNAm of BECs collected from oral swabs, rather than DNAm in neural tissues, where biobehavioral patterns of response originate. DNAm is tissue‐specific in nature, and there are clearly many differences among the epigenetic marks measured in buccal epithelium and brain tissue. Current research in epigenetics is elucidating the level of DNAm concordance between brain and tissues/fluids commonly collected for DNA analysis, including saliva, BECs, and white blood cells (Edgar, Jones, Meaney, Turecki, & Kobor, 2017; Farré et al., 2015; Smith et al., 2015). Although such research is ongoing, emergent literature suggests that DNAm located in specific genomic regions, such as CpG islands within gene coding regions, may be more highly conserved across tissues and therefore potentially informative of brain DNAm patterns (Edgar et al., 2017; Walton et al., 2016).

Our results reveal age‐related DNAm changes from age 15 to 18 in *DLX5*,* IGF2*,* PRUNE2,* or *MYO16*, consistent with prior research identifying increased DNAm changes in brain tissue and blood from childhood to adolescence as compared to more minimal changes that occur across adulthood (Alisch et al., 2012; Lister et al., 2013). However, given the confounding of DNAm processing technology with age in this study, we cannot rule out changes in DNAm between age 15 and age 18 resulted from differences between the 27K array and 450K technologies and differences in data processing and normalization. Additionally, differences in the cell composition of buccal samples between age 15 and age 18 may have also contributed. A different study design will be required to test broadly how age is reflected in DNAm changes. Finally, to test our hypotheses related to longitudinal stability, we conducted, by necessity, a more constrained analysis of DNAm at age 18, though future analyses will explore the full range of data collected at 18 in relation to both early environmental and biobehavioral reactivity factors.

These limitations notwithstanding, the present study's integration of pertinent information from different biological arenas (e.g., epigenetics, autonomic physiology) with psychological constructs, is consistent with a new strategy in mental health research proposed by the National Institute of Mental Health (NIMH). The Research Domain Criteria (RDoC) framework guides mental health research to recognize the broader biological and psychological contexts of health and behavior and to understand the phenotypic variability within clinical disorders (Morris & Cuthbert, 2012; Simmons & Quinn, 2013). As such, advancing investigations into the relations among epigenetic differences, childhood behavior problems, and patterns of stress responsivity could support earlier identification of inauspicious developmental and mental health outcomes.

In conclusion, this study revealed strong, prospective associations of observational measures of childhood inhibition/disinhibition with patterns of DNAm in BECs harvested at both mid‐adolescence and early adulthood. Though current analyses do not allow for firm inferences of antecedence and causality, such associations focus attention upon possible linkages between inhibition/disinhibition dimensions of children's temperament and behavior and the *DLX5* and *IGF2* genes that have diverse developmental and regulatory functions. Our findings offer provisional evidence for a developmental, epigenetic biomarker of internal biobehavioral response predilections. As with many other developmental processes, epigenetic modifications integrate the complex interactions of environmental context and constitutional biology, providing insight into developmental trajectories and long‐term health outcomes.

## AUTHOR CONTRIBUTIONS

S.J.G. performed all analyses and wrote the paper with D.S.R.; M.P. contributed to DNA methylation data generation and to writing the paper; E.E. and P.F. gave technical support and conceptual advice; M.J.E., C.H., M.S.K., and W.T.B. conceived of the project and supervised; M.J.E. conceived of the original cohort study and acquired all behavioral and psychological data. All authors discussed the results and implications at all stages of analysis and commented on and approved of the manuscript.
