# Supplementary material for: Children's biobehavioral reactivity to challenge predicts DNA methylation in adolescence and emerging adulthood
Source: Dev Sci. 2018 Sep 21;22(2):e12739. doi: 10.1111/desc.12739 (PMC6433477; doi:10.1111/desc.12739)
Supplement: Supplementary file 1 [file DESC-22-e12739-s001.pdf]

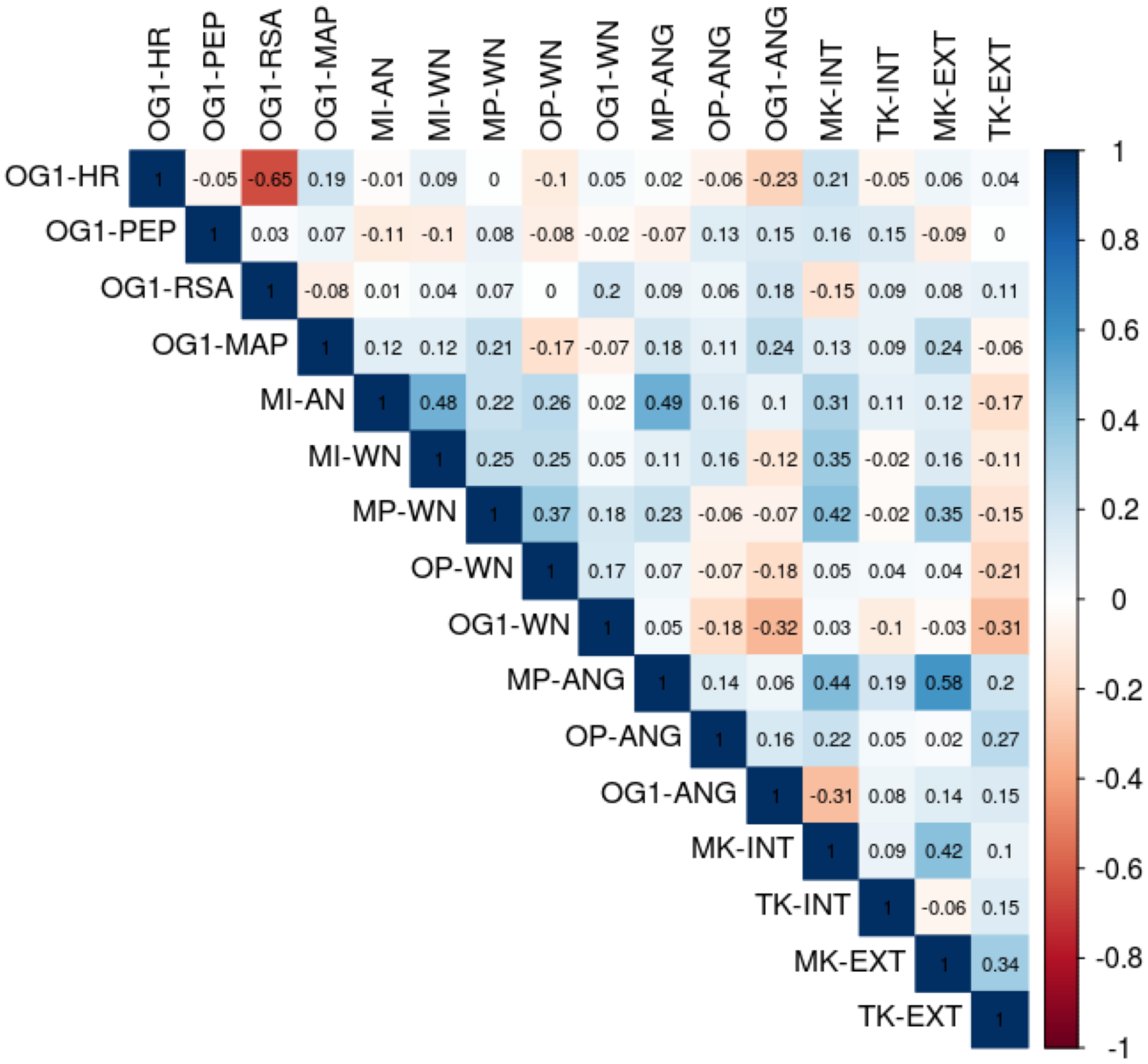

Supplementary Figure 1 Associations between temperament, mental health and ANS measures supported preexisting relationships between these traits. Correlation matrix of pairwise spearman correlations run on 16 original traits (*n*= 55).

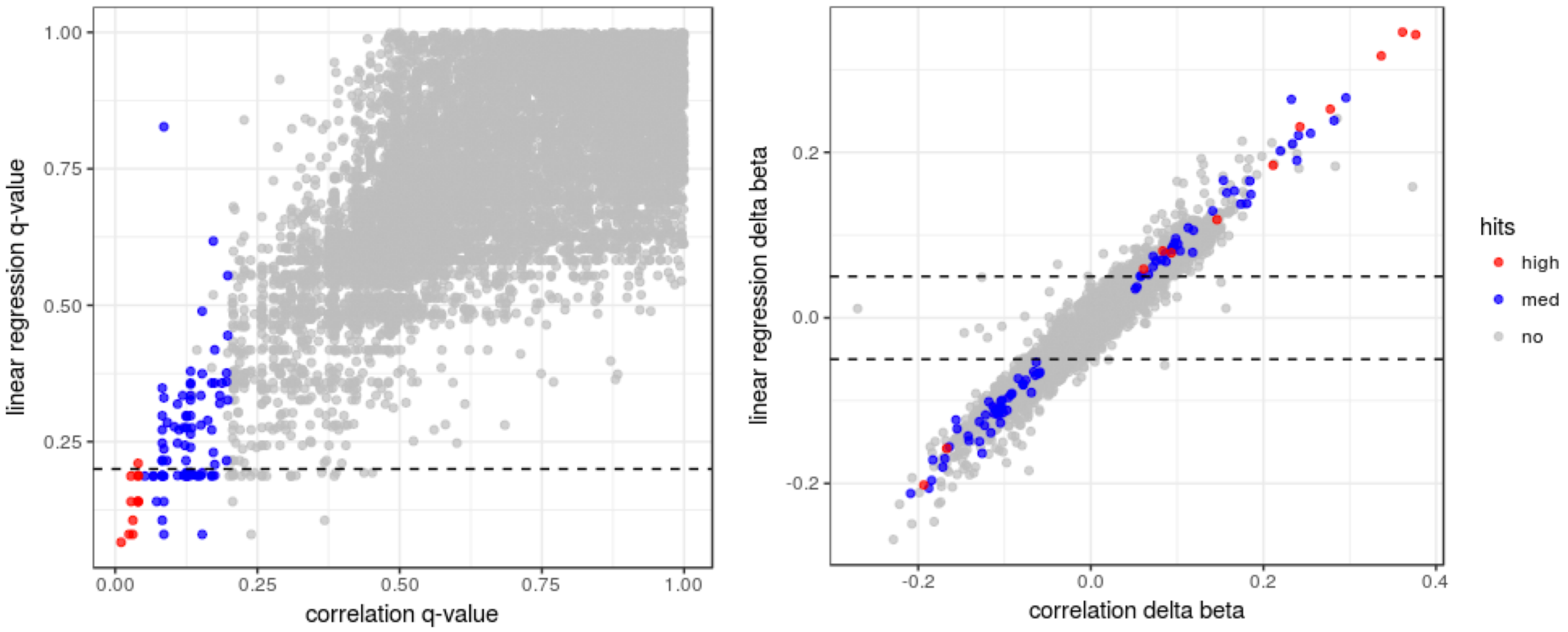

Supplementary Figure 2 Comparison of FDR corrected *p*-values (*q*-values) and effect sizes (delta betas) from Spearman correlations and linear regressions. In left panel, Spearman *q-values* vs. *q-values* produced by linear regression covaried by sex and minority status. In right panel, delta betas from Spearman correlation analysis vs. linear regression. Of the 93 high and medium confidence CpGs, 40 were retained at a *q*-value < 0.2; this included 11 of the 12 original high confidence CpGs and 29 of 81 medium confidence. Horizontal lines represent thresholds used for medium confidence sites (*q-value* < 0.2, absolute db > 0.05). Sites are colored by their significance in Spearman analysis (red = high confidence, blue = medium confidence, grey = not significant) (*n*= 55).

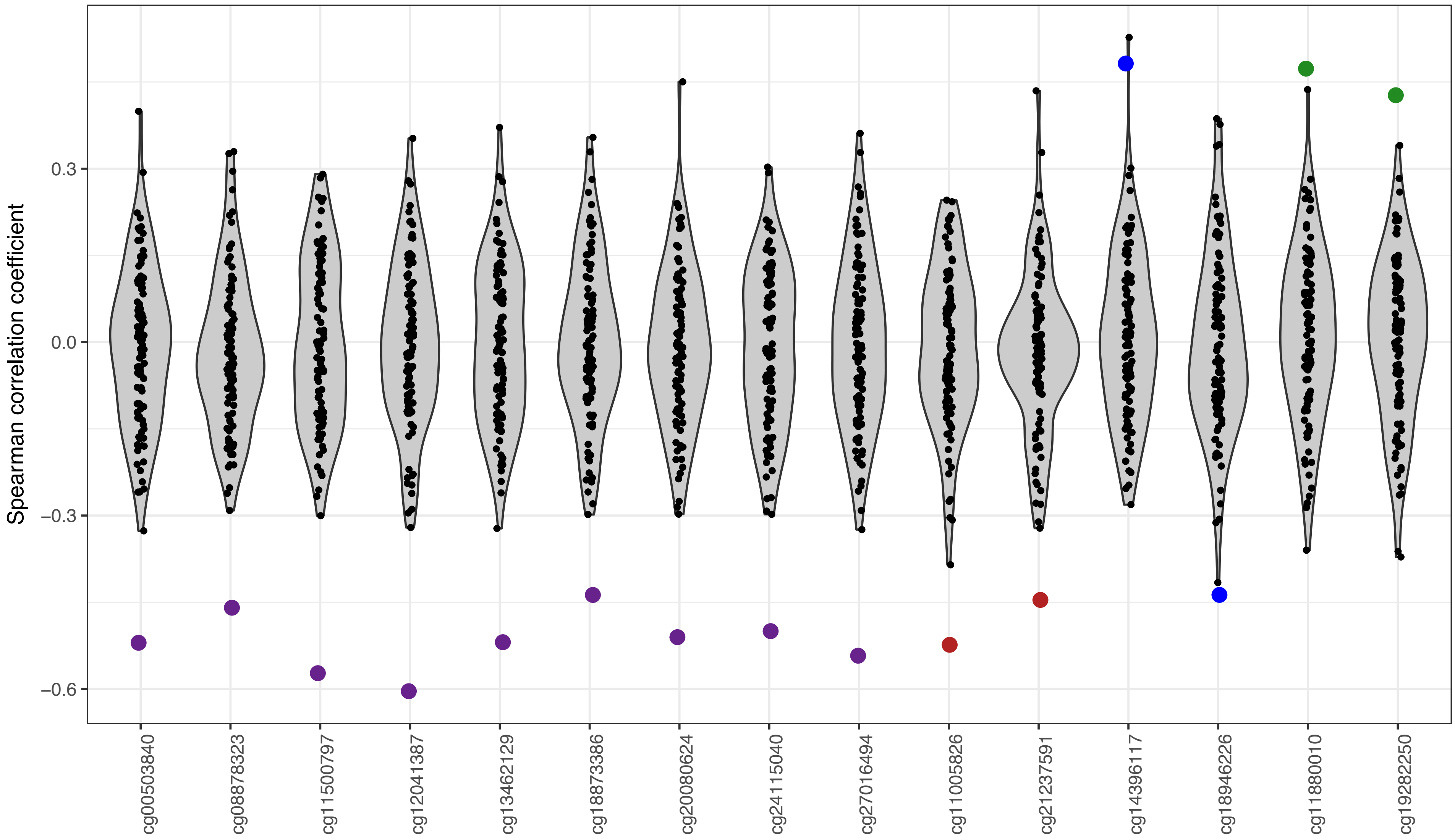

Supplementary Figure 3 Associations between Inhibition/Disinhibition scores and *DLX5* differentially methylated CpGs passed permutation tests at age 15. Inhibition/Disinhibition scores were randomly assigned 100 times and correlated with DNA methylation of the respective differentially methylated CpGs at age 15. True coefficients fell outside of the 99 percentile of the null distributions suggesting that these associations were not spurious. Colors represent to which gene the CpG is mapped: purple = *DLX5*, red= *IGF2*, blue= *MYO16*, green = *PRUNE2*(*n*= 55).

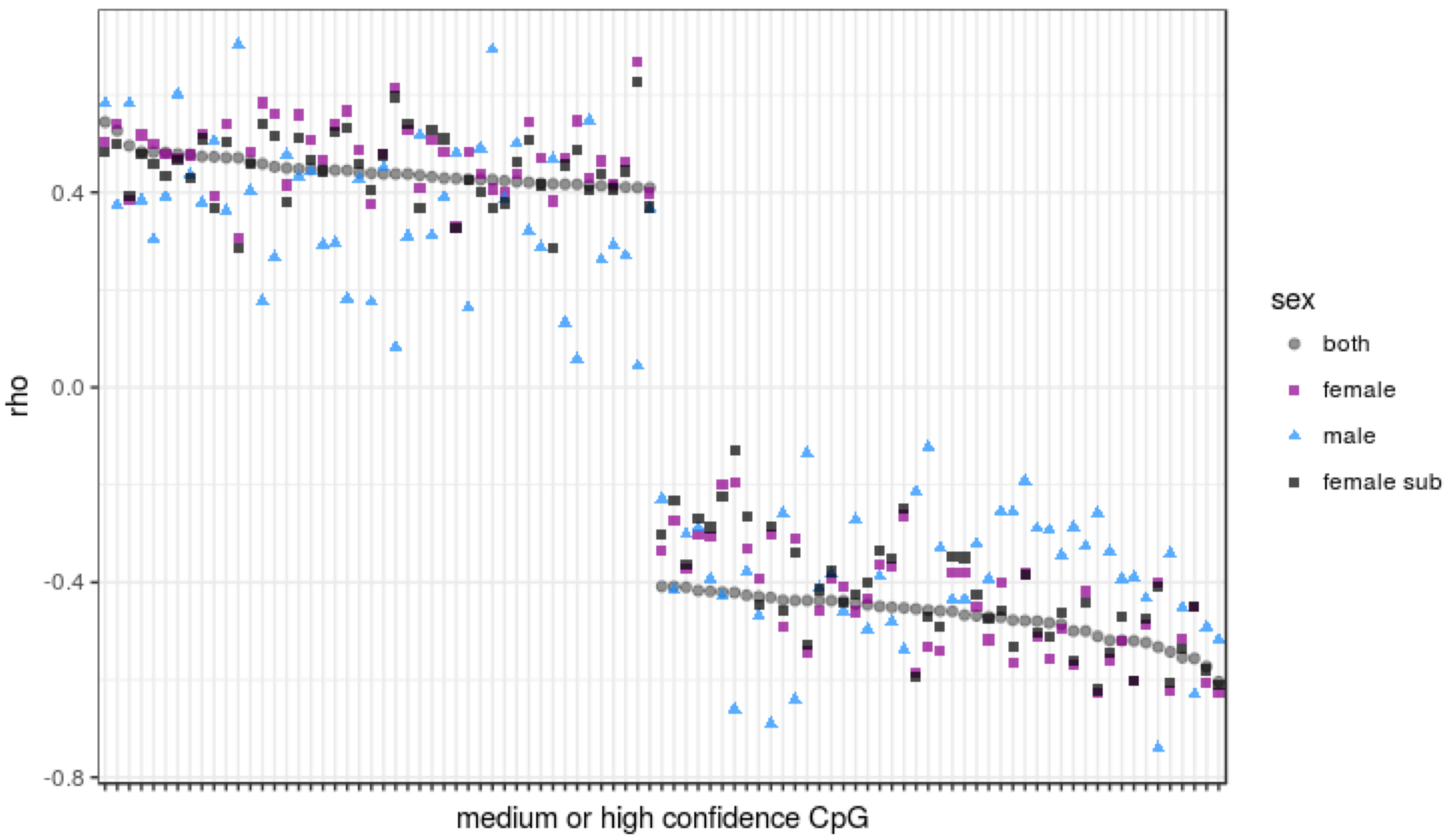

Supplementary Figure 4 Correlations between DNA methylation at age 15 and Biobehavioural Inhibition/Disinhibition in males, females, subsampled females. Difference in magnitude in correlation coefficient between males and females was not greatly altered by sample size in high and medium confidence CpGs. Full cohort (gray circle, n=55), females only (pink square, n=36) and males only (blue triangle, n=19). Average of subsampled females to n=19 and running correlations 100 times in black squares.

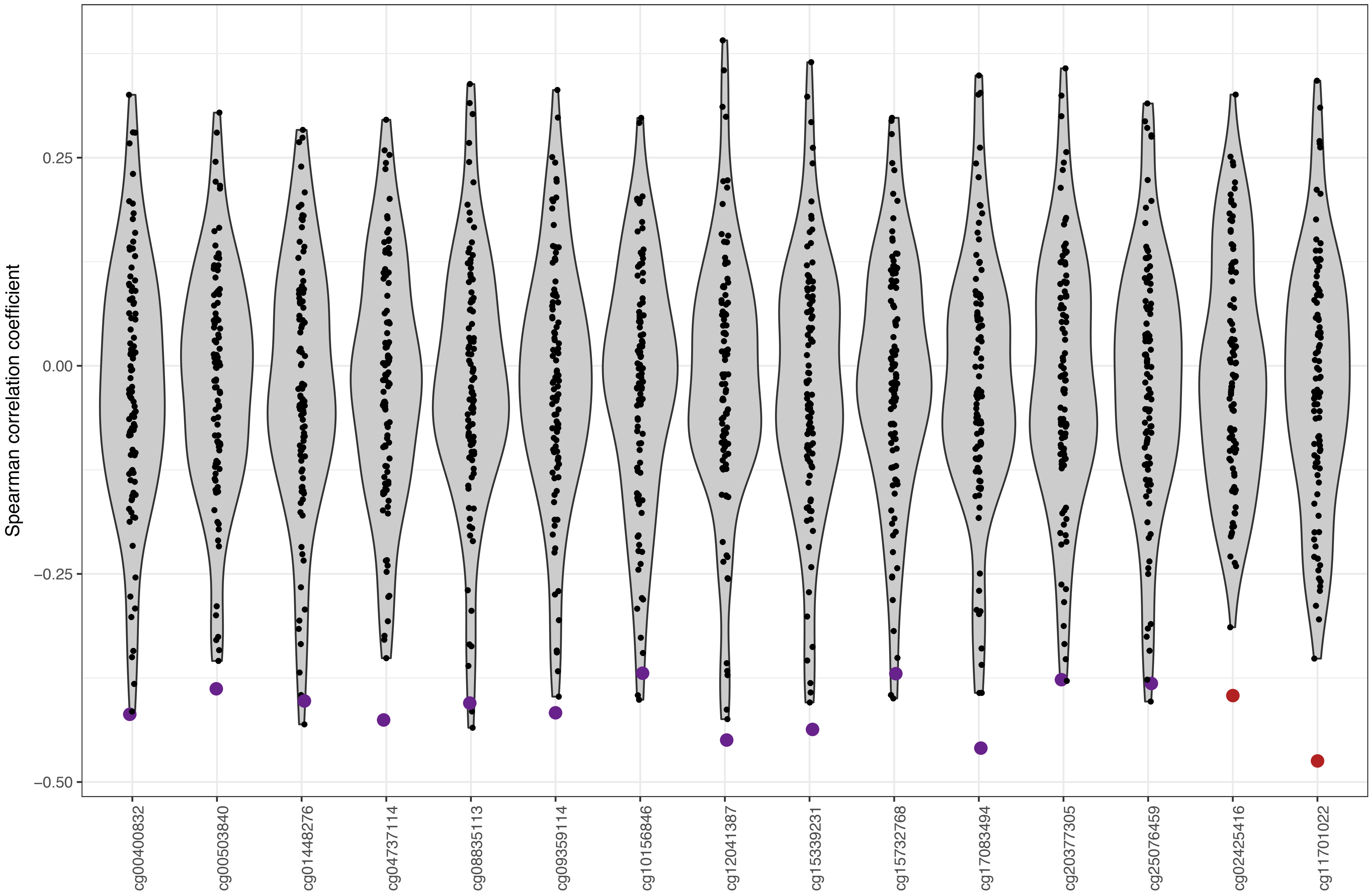

Supplementary Figure 5 Associations between Inhibition/Disinhibition scores and *DLX5* differentially methylated CpGs passed permutation tests at age 18. Inhibition/Disinhibition scores were randomly assigned 100 times and correlated with DNA methylation of the respective differentially methylated CpGs at age 18. True coefficients fell outside of the 97 percentile of the null distributions suggesting that these associations were not spurious. Colors represent to which gene the CpG is mapped: purple = *DLX5*, red= *IGF2 (n*= 52).

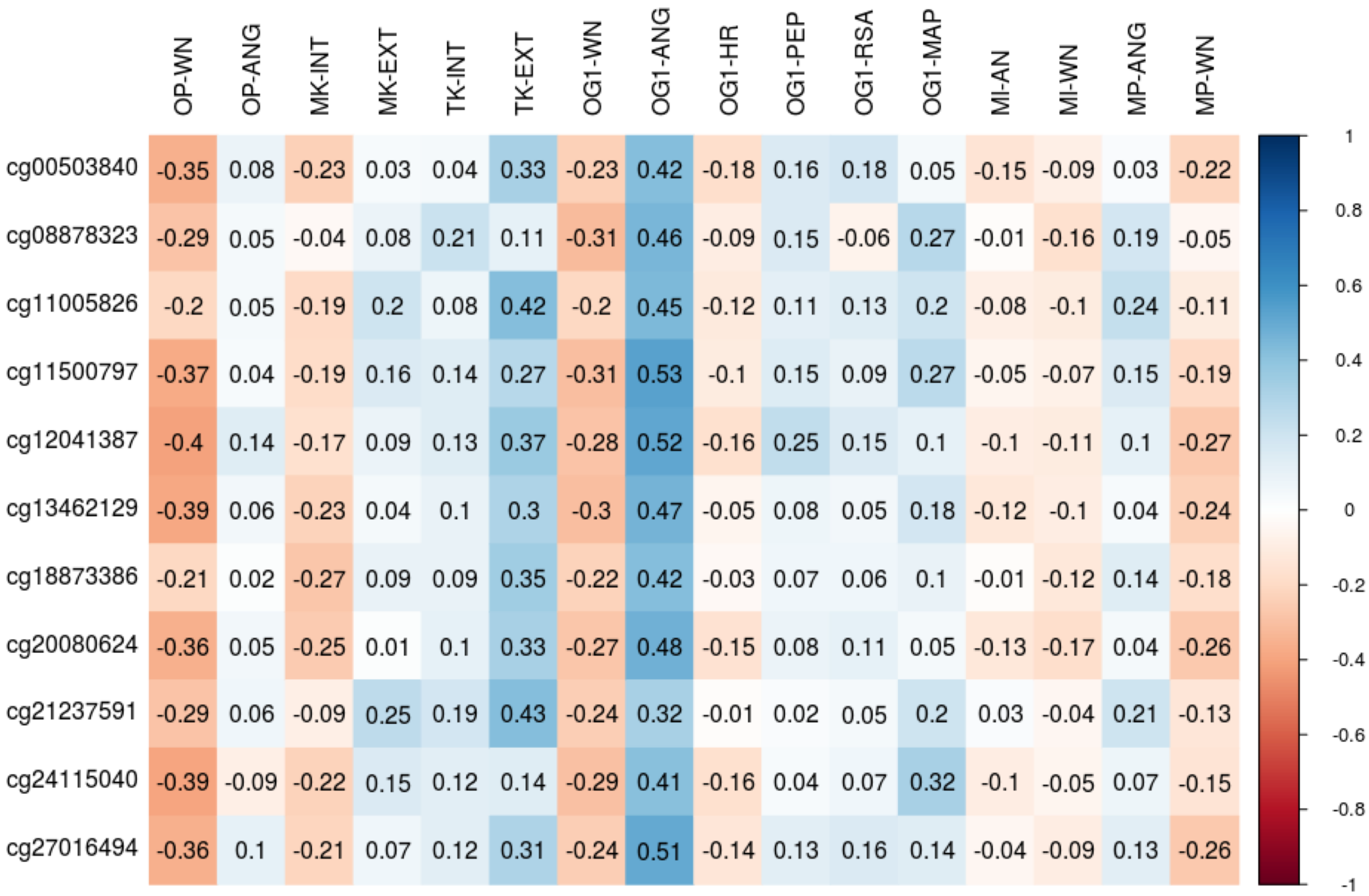

Supplementary Figure 6 Associations between measures underlying Inhibition/Disinhibition and differentially methylated CpGs in *DLX5* and *IGF2*. Observational anger measured at grade 1 was the most strongly correlated with DNA methylation at both genes.
